# Supplementary figures and images for: Integrating Genomics and Transcriptomics to Identify Candidate Genes for Egg Production in Taihe Black-Bone Silky Fowls (Gallus gallus domesticus Brisson)
Source: Int J Mol Sci. 2024 Aug 29;25(17):9373. doi: 10.3390/ijms25179373 (PMC11395579; doi:10.3390/ijms25179373)

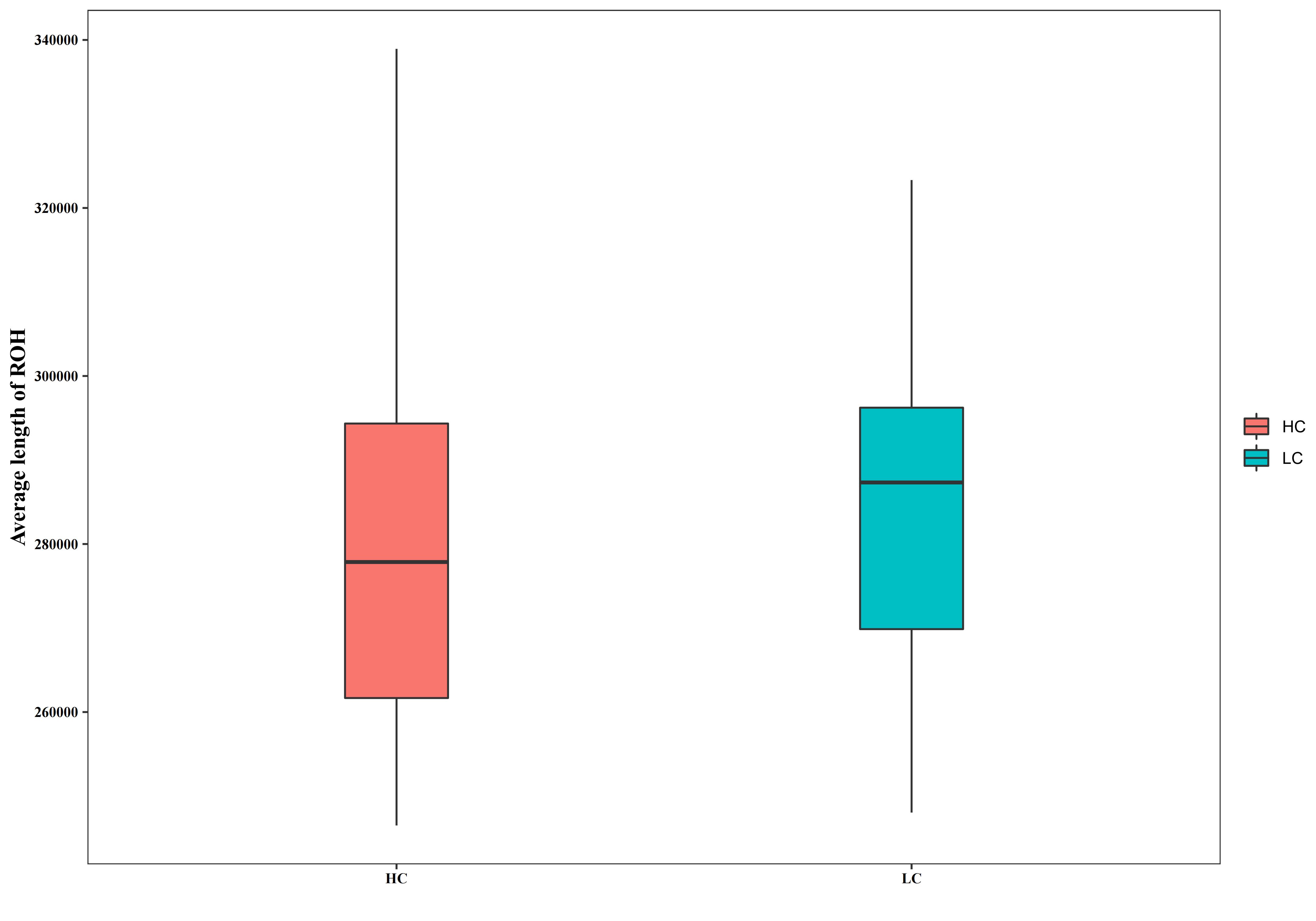

Supplement: Supplementary file 1 [file ijms-25-09373-s001.zip › Figure S3.jpg]

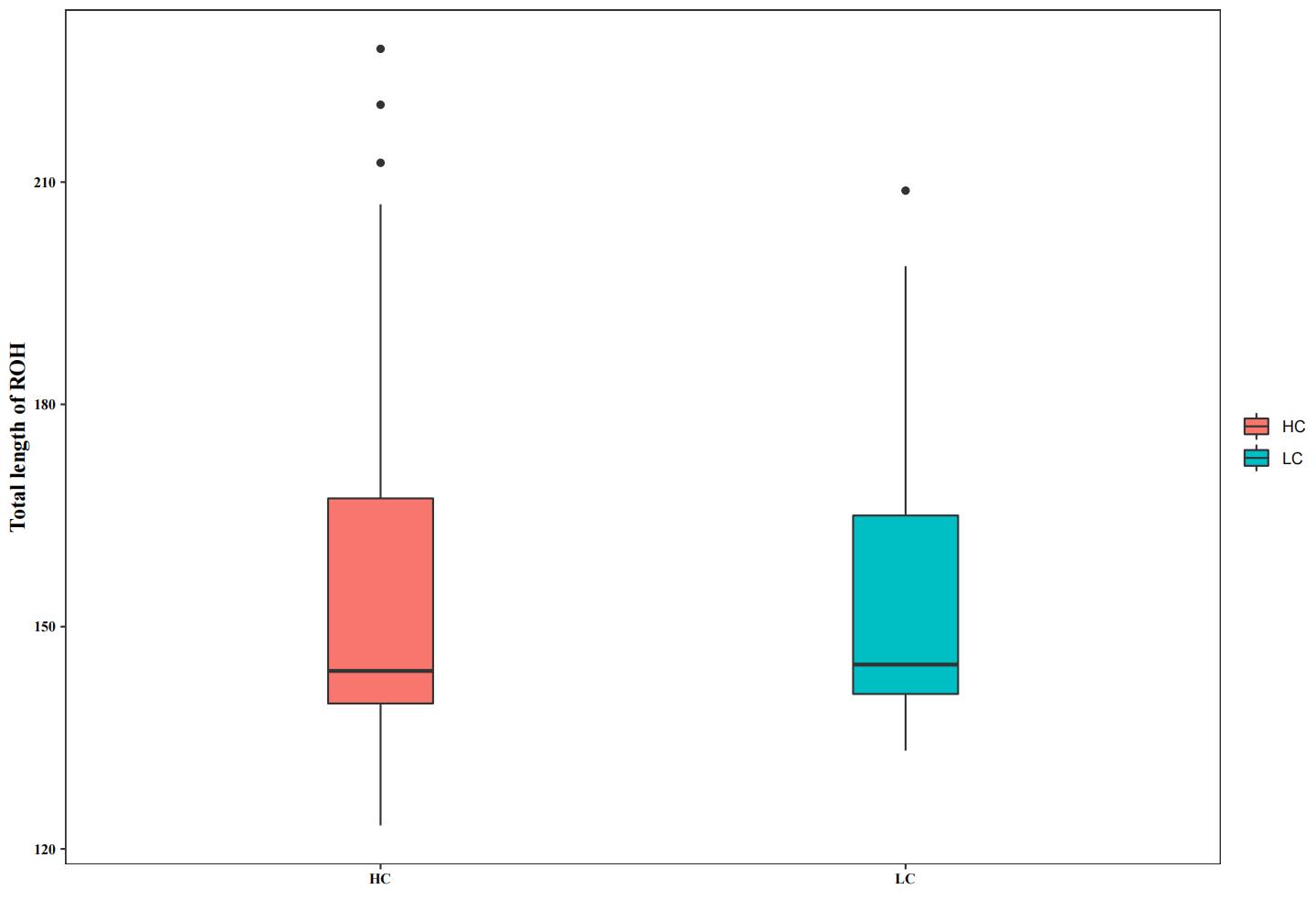

Supplement: Supplementary file 1 [file ijms-25-09373-s001.zip › Figure S4.jpg]
